# Fabrication Technology and Characteristics Research of the Acceleration Sensor Based on Li-Doped ZnO Piezoelectric Thin Films

**DOI:** 10.3390/mi9040178

**Published:** 2018-04-12

**Authors:** Sen Li, Xiaofeng Zhao, Yinan Bai, Yi Li, Chunpeng Ai, Dianzhong Wen

**Affiliations:** Key Laboratory of Electronics Engineering, College of Heilongjiang Province, Heilongjiang University, Harbin 150080, China; 2161311@s.hlju.edu.cn (S.L.); 2141207@s.hlju.edu.cn (Y.B.); 2171256@s.hlju.edu.cn (Y.L.); 2011026@hlju.edu.cn (C.A.); wendianzhong@hlju.edu.cn (D.W.)

**Keywords:** acceleration sensor, piezoelectric thin films, Li-doped ZnO thin films, MEMS technology

## Abstract

An acceleration sensor based on piezoelectric thin films is proposed in this paper, which comprises the elastic element of a silicon cantilever beam and a piezoelectric structure with Li-doped ZnO piezoelectric thin films. The Li-doped ZnO piezoelectric thin films were prepared on SiO_2_/Si by radio frequency (RF) magnetron sputtering method. The microstructure and micrograph of ZnO piezoelectric thin films is analysed by a X-ray diffractometer (XRD), scanning electron microscope (SEM), X-ray photoelectron spectroscopy (XPS), and piezoresponse force microscopy (PFM), respectively. When the sputtering power of 220 W and Li-doped concentration of 5%, ZnO piezoelectric thin films have a preferred (002) orientation. The chips of the sensor were fabricated on the <100> silicon substrate by micro-electromechanical systems (MEMS) technology, meanwhile, the proposed sensor was packaged on the printed circuit board (PCB). The experimental results show the sensitivity of the proposed sensor is 29.48 mV/g at resonant frequency (1479.8 Hz).

## 1. Introduction

ZnO is a typical n-type compound semiconductor. ZnO thin films have many advantages, such as wide band gap (Eg ≈ 3.37 eV), high visible light transmittance, good ultraviolet absorption, and high electrical conductivity, etc. [1,2,3,4,5,6]. In 2008, S.H. Jeong, et al. prepared Lithium-doped ZnO (LZO) films which were deposited by a radio frequency (RF) magnetron sputtering method using a Li-doped ZnO ceramic target with various ratios (0 to 10 wt% LiCl dopant). They studied the structural, optical, and electrical properties of the LZO films by X-ray diffractometer (XRD), atomic force microscope (AFM), scanning electron microscope (SEM), X-ray photoelectron spectroscopy (XPS), and 4-point probe analyses. The experimental results show that LZO films with 4 wt% are apposite for piezoelectrical application [7]. In 2012, T.A. Johny, et al. prepared ZnO thin films (thickness is 2.0 μm) of undoped and lithium-doped ZnO on Pt/Ti/SiO_2_/Si substrate by sol-gel method. Lithium impurity is used to improve the electrical resistivity as well as the preferred *c*-axis orientation. The experimental results show that the transverse piezoelectric coefficient *d*_31_ of (Zn_0.95_Li_0.05_)O thin films reaches −0.41 C/m^2^ and a displacement of 1.5 nm/V [8]. In 2015, M. Laurenti, et al. fabricated the piezopolymeric transducer (PPT) for health monitoring systems in space applications. The transducer consists of a *c*-axis-oriented ZnO thin film coupled to a pair of flexible polyimide foils coated with gold (Au) electrodes. At room temperature (RT), the ZnO thin films and top electrode were deposited on the Au/polyimide foils by the RF magnetron sputtering method. The experimental measured piezoelectric coefficient *d*_33_ value is 12.3 pm/V [9]. In 2017, V.V. Sasi, et al. prepared performed *c*-axis oriented ZnO films on top of Si(111) substrates with a 3C-SiC(111) epitaxial buffer layer by RF magnetron sputtering method. They researched the effect of the different O_2_/Ar ratios on the ZnO (002) crystal orientation. The experimental results show that the full width at half maximum (FWHM) values are uniform when the O_2_/Ar ratios are 30% to 60%, and the post-annealing temperature of 600 °C is the most optimized range for the ZnO (002) deposition [10]. Up to now, ZnO piezoelectric thin films were prepared by different technology methods. The influence of the doping impurity atom (such as Li, Mn and Al, etc.) on properties of thin films has been researched in detail, and the results show that the doping method can obtain a preferred c-axis orientation of the ZnO thin films. Meanwhile, the impedance and piezoelectric properties of the ZnO thin films could be improved.

In this study, we prepared the Li-doped ZnO thin films by the RF magnetron sputtering method. Based on the analysis about the microstructure and piezoelectric property of the proposed thin films, the acceleration sensor with piezoelectric structure was fabricated and packaged on silicon substrate by micro-electromechanical systems (MEMS) technology. Meanwhile, the characteristics of the fabricated sensor was researched in this paper.

## 2. Basic Structure and Operation Principle

### 2.1. Basic Structure

**Figure 1:**
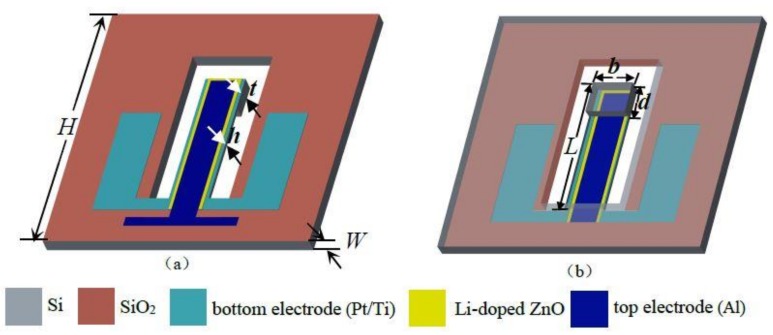
Basic structure of the acceleration sensor: (**a**) front view; (**b**) back view.

Figure 1a,b show the front and back view of the Li-doped ZnO piezoelectric thin films acceleration sensor, which consists of the silicon elastic cantilever beam and piezoelectric structure (Al/Li-doped ZnO/Pt/Ti). The cantilever beam structure is comprised of the silicon cantilever beam and a mass block on the free end of the beam. The length, width and thickness of the cantilever beam are *L*, *b* and *h*, respectively. *b* is the length of the mass, *d* is the width and *t* is the thickness. The piezoelectric structure has three layers, include the bottom electrode layer of the Pt/Ti, Li-doped ZnO piezoelectric thin films, and the top electrode layer of the Al. The area of the sensor chip is 10 mm × 10 mm. The area of the piezoelectric thin films is 6300 μm × 800 μm. 

### 2.2. Operation Principle

**Figure 2:**
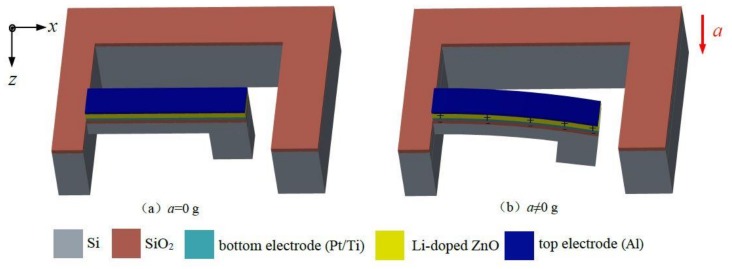
The operating principle of the Li-doped ZnO piezoelectric thin films acceleration sensor.

Figure 2 shows the operating principle of the Li-doped ZnO piezoelectric thin films acceleration sensor. As shown in Figure 2a, without considering the acceleration of gravity, when the external acceleration *a* = 0 g, the elastic beam does not move. As shown in Figure 2b, when the external acceleration *a* is applied to the proposed sensor along the *z*-axis direction, according to the Newton’s second law, the mass produced concentrated force *F* which acts on the beam, as:

(1) F=ma

where *m* is the mass of the mass block. 

Under the acts of the *F*, the cantilever beam is deformed elastically. According to the mechanics analysis of the cantilever beam and the small deflection theory [11], when the *F* acts on the beam, the longitudinal stress $\sigma_{l}$ of the surface of the elastic beam along the *x-*axis is:

(2) $$\sigma_{l}=\sigma_{1}=\frac{6F}{bh^{2}}\cdot L$$

Based on the piezoelectric effect, the two surfaces (top and bottom) of the Li-doped ZnO piezoelectric films appear positively and negatively charged along the *z*-axis, the charge amount $q_{3}$ can be obtained:

(3) $$q_{3}=d_{31}\sigma_{1}$$

where *d*_31_ is piezoelectric coefficient, $\sigma_{1}$ is the normal stress along the *x*-axis.

According to the definition, the capacitance *C* between the top and bottom electrode of the piezoelectric structure can be given:

(4) $$C=\frac{q_{3}}{V}$$

where *V* is the output voltage between the top and bottom electrode.

By substituting Equation (3) into (4), *V* can be obtained:

(5) $$V=d_{31}\cdot \frac{6mL}{bh^{2}C}\cdot a$$

By definition, combined with the Equation (5), so the sensitivity *S* of the sensor can be expressed as:

(6) $$S=\frac{V}{a}=d_{31}\cdot \frac{6mL}{bh^{2}C}$$

On the basis of a theoretical analysis for the silicon cantilever beam and piezoelectric effect, the proposed structure can achieve the acceleration measurement, meanwhile, the sensitivity of the sensor is proportional to the piezoelectric coefficient *d*_31_ when the size (*L* = 6000 μm, *b* = 1500 μm, *h* = 60 μm, *d* = 1000 μm, *t* = 465 μm) of the cantilever beam is determined.

## 3. Fabrication Technology

**Figure 3:**
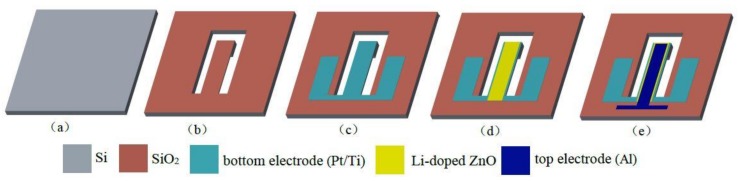
The main fabrication technology process of the proposed sensor: (**a**) the cleaning of the silicon wafer; (**b**) the releasing of the cantilever beam; (**c**) the growing of the Pt/Ti bottom electrode layer; (**d**) the sputtering of the Li-doped ZnO piezoelectric layer; (**e**) the evaporating the of Al top electrode layer.

Figure 3 shows the main fabrication process of the proposed sensor chip. Based on MEMS technology and the mask technique, the main steps are: (a) The cleaning the n-type <100> silicon wafer with double-sided polishing; (b) The first oxidation, growing the SiO_2_ layer on the silicon substrate by using a thermal growth method and the thickness of the SiO_2_ layer is about 300 nm. The first photolithography, etching the back of the chip using an inductively coupled plasma (ICP), meanwhile forming a silicon cup structure. The second lithography, etching the front of the chip, the releasing the cantilever beam, and formats a cantilever beam structure with a mass block; (c) The growing the Pt/Ti multi-layer films by using magnetron sputtering (JGP-DZS, Shenyang Sky Technology Development Co. Ltd., Shenyang, China) and forming the bottom electrode using the Cu foil mask method; (d) The sputtering the Li-doped ZnO piezoelectric thin films by using an RF magnetron sputtering system with high vacuum and forming the piezoelectric layer; (e) The evaporating the Al by using vacuum deposition (ZZ-450A, Beijing Beiyi Innovation Vacuum Technology Co. Ltd., Beijing, China) and as the top electrode.

**Figure 4:**
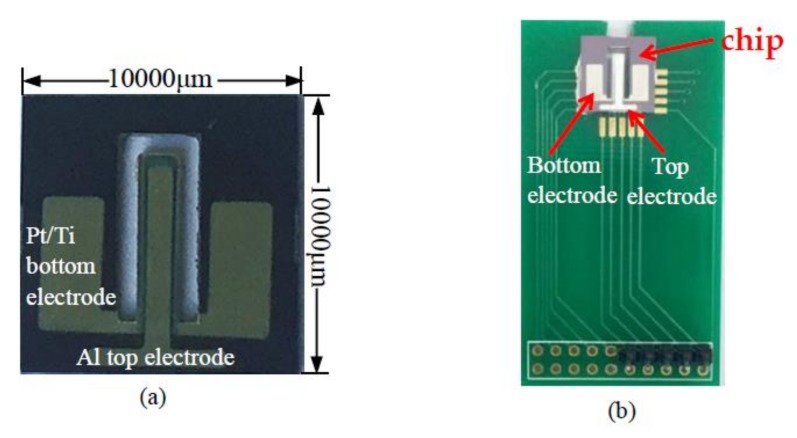
The chip photos of acceleration sensor: (**a**) photo of chip; (**b**) photo of packaging chip.

The photograph of the completed acceleration sensor chip is shown in the Figure 4a. The main steps of the chip packaging process are: First, the chip was pasted onto the printed circuit board (PCB) by using a seal adhesive (730 RTV Sealant, Dow Corning Corporation, Midland, MI, USA) and then dried in a drying box at 80 °C for 3 h. Second, the electrodes of the chip were ultrasonically welded to connect to the electrode pads of the PCB by using a high purity gold wire (purity: 99.99%) at the integrated chip press welder (KNS4526, Kullicke & Soffa, Haifa, Israel). Figure 4b presents photos of the packaging chip.

## 4. Results and Discussion

### 4.1. XRD Analysis 

**Figure 5:**
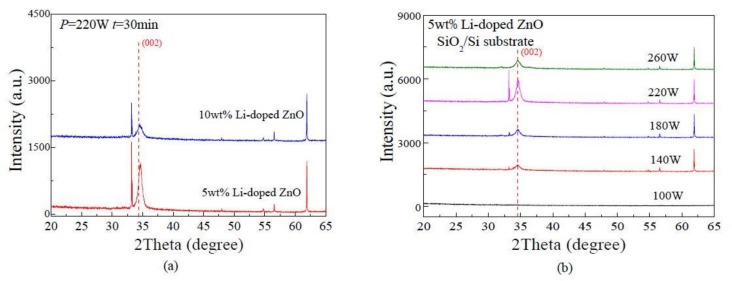
The X-ray diffractometer (XRD) patterns of the Li-doped ZnO thin films: (**a**) under different Li doping concentrations; (**b**) under different sputtering powers.

The crystal structure of the Li-doped ZnO thin films is characterized by XRD (Bruker AXS D8 ADVANCE, Bruker Corporation, Karlsruhe, Germany). The Li-doped ZnO piezoelectric thin films are prepared on a SiO_2_/Si substrate by RF magnetron sputtering, the deposition temperature, the deposition time, and the working pressure were 200 °C, 30 min and 1.0 Pa, respectively. Figure 5a presents the XRD patterns of different Li-doped concentrations (5 wt%, 10 wt%) under the sputtering power of 220 W. With the lithium impurity concentration increases, the (002) diffraction peak intensity gradually decreases, and when the FWHM value gradually increases, the (002) peak moves towards higher diffraction angle with the increase of doping concentration. Meanwhile the peak position is shifted to the right, indicating that the degree of ZnO crystallization decreases with the doping concentration, which is in agreement with the XRD patterns proposed by P. Chand, et al. [12]. When the impurity concentration reaches 10 wt%, the (002) peak is almost invisible. In summary, the Li-doping concentration of 5 wt% is the most appropriate. When deposition temperature, the deposition time, and the working pressure were 200 °C, 30 min and 1.0 Pa, we studied the effects of the sputtering power on the microstructure of thin films. Figure 5b presents the XRD patterns of 5 wt% Li-doped ZnO thin films deposited with various sputtering powers (100 W, 140 W, 180 W, 220 W, 260 W) on a SiO_2_/Si substrate. The peak of 220 W shows a better crystal orientation, and has a good crystallinity.

### 4.2. XPS Analysis 

**Figure 6:**
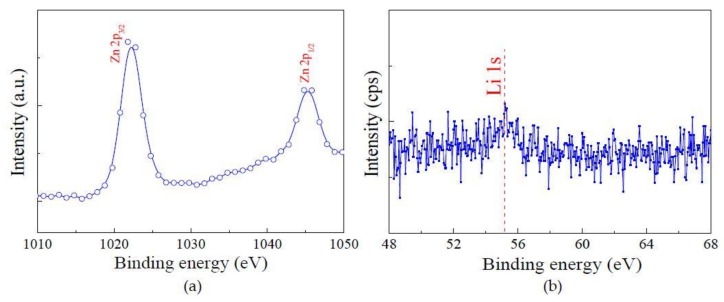
X-ray photoelectron spectroscopy (XPS) spectra of 5 wt% Li-doped ZnO thin films (**a**) XPS spectra for Zn 2p; (**b**) narrow scan at Li 1s peak.

Figure 6 presents the chemical bonding states of 5 wt% Li-doped ZnO thin films by X-ray photoelectron spectroscopy (XPS, VG ESCALAB MK II, VG Instruments, Manchester, UK), with the scan range from 0 to 1350 eV. As shown in Figure 6a, the binding energy of Zn 2p_3/2_ and Zn 2p_1/2_ are located around 1021.8 eV, 1044.8 eV, respectively. The peak intensity of the Zn 2p_3/2_ state is relatively higher that the Zn 2p_1/2_, clearly prove that the Zn ions in the thin films are mainly in the chemical state of Zn^2+^, and the Zn^0^ are correspondingly less [13,14,15]. Figure 6b shows the Li 1s spectra of 5 wt% Li-doped ZnO thin films. The Li 1s peak was observed at around 55.3 eV, the binding energy of Li 1s core level is in the range 52–56 eV [14,15,16]. The quantitative analysis result shows that the weight percentage of Li elements is 4.36 wt% [17].

### 4.3. SEM Analysis

**Figure 7:**
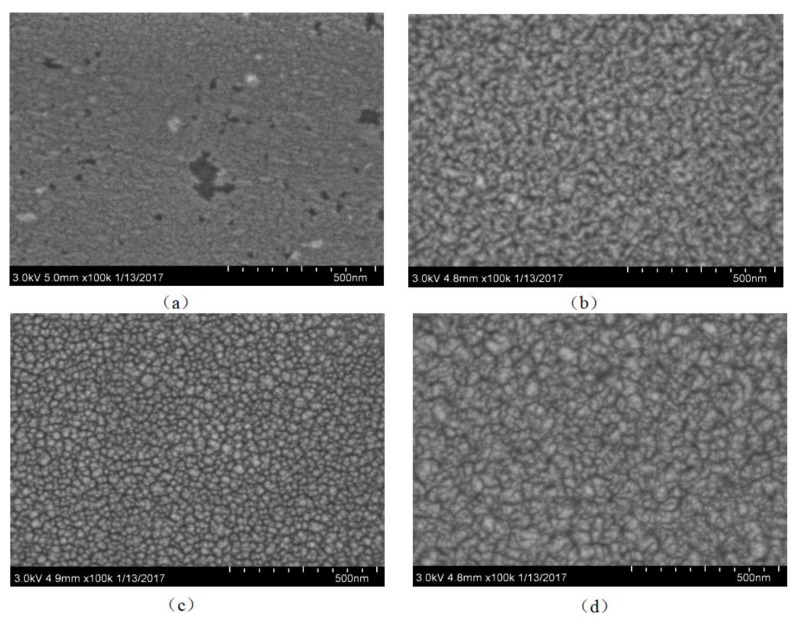
Scanning electron microscope (SEM) images of the Li-doped ZnO thin films under different sputtering powers: (**a**) 140 W; (**b**) 180 W; (**c**) 220 W; (**d**) 260 W.

The surface morphology of the thin films are analyzed by using SEM (SU8010, Hitachi Limited, Tokyo, Japan). When the doping concentration, deposition time, working pressure and deposition temperature are fixed at 5 wt%, 30 min, 1.0 Pa and 200 °C, we analyzed the effect of sputtering power on morphology. Figure 7a–d show the SEM images of the Li-doped ZnO thin films with different sputtering powers. Figure 7a shows the surface morphology of thin films for 140 W, the thin films quality is poor. Figure 7c presents that the surface morphology of thin films for 220 W has a good grain size uniformity and the grains are dense and spherical. From Figure 7, we can see that the thin films size increases first and then reduce with the sputtering power. When the sputtering power is 220 W, the grain uniformity of the films is better, and there are no obvious holes, gaps and wrinkles.

### 4.4. Piezoresponse Force Microscopy (PFM) Analysis

**Figure 8:**
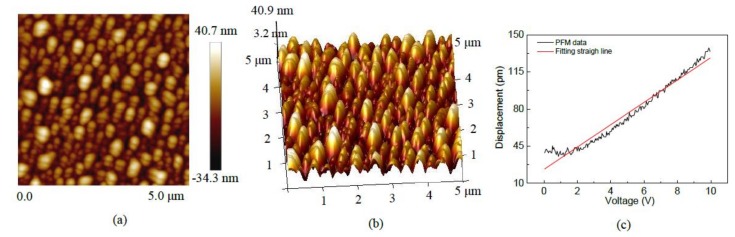
Piezoresponse force microscopy (PFM) images of 5 wt% Li-doped ZnO thin films: (**a**) the two-dimensional topography; (**b**) the three-dimensional topography; (**c**) the relationship curve between stylus tip displacement and excitation voltages.

Figure 8a,b show that the two-dimensional (2D) and three-dimensional (3D) topographies of 5 wt% Li-doped ZnO thin films. The images of the thin films was obtained and characterited by piezoresponse force microscopy (PFM, Bruker Multimode8, Bruker Corporation, Karlsruhe, Germany). The area of the scanning region is 5 μm × 5 μm. The topography images show that the grains are uniformly distributed on the surface of the thin films, and there are no obvious defects such as cracks or scratches. Figure 8c shows the relationship curve between stylus tip displacement and the excitation voltages. The cantilever beam tip plated on the surface of the thin films, and the excitation voltage between the conductive cantilever beam tip and the bottom electrode of the sample, from 0 to 10 V. Via analysis of the relationship curve between the displacement of the PFM cantilever beam tip and the excitation voltages, we calculated that the piezoelectric coefficient *d*_33_ is 10.55 pm/V [18].

### 4.5. Characteristics of the Acceleration Sensor

**Figure 9:**
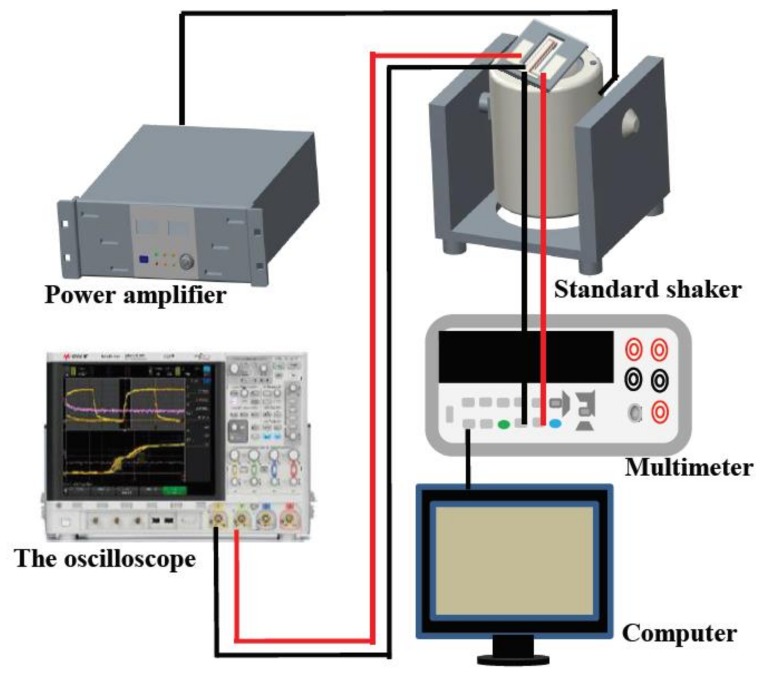
The testing system of the acceleration sensor.

Figure 9 shows that the testing system of the acceleration sensor, which consists of the standard vibration table (Dongling ESS-050, Dongling Vibration Test Instrument Co., Ltd, Suzhou, China), power amplifier (Dongling PA-1200B, Dongling Vibration Test Instrument Co., Ltd, Suzhou, China), oscillograph (Agilent DSO-X 4145A, Agilent Technologies Inc., Santa Clara, CA, USA) and multimeter (Agilent 34401A, Agilent Technologies Inc., Santa Clara, CA, USA). The system can be tested for vibration characteristics and sensitive characteristics (exciting frequency from 50 to 20,000 Hz, acceleration from 0 to 30 g), meanwhile it can achieve data acquisition automatically. 

**Figure 10:**
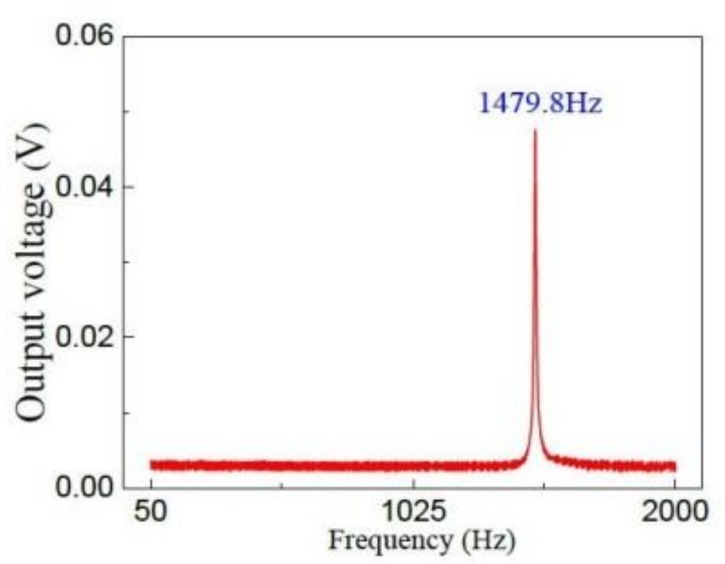
The relationship curve between output voltage and excitation frequencies.

The dynamic characteristics of the proposed sensor was tested by using a vibration characteristics testing module. We set the excitation frequency (from 50 to 2000 Hz) and studied the effect of frequency on output voltage between two electrodes (top and bottom). The chip of the acceleration sensor is attached on the surface of the standard shaker, the external acceleration *a* acts on the sensor along the *z*-axis. Figure 10 shows that the relationship curve between output voltage and excitation frequencies of the proposed sensor when the excitation acceleration *a* is 1.5 g. The experimental results show that the natural frequency (resonant frequency) of the sensor is 1479.8 Hz. When the excitation frequency is less than 1479.8 Hz or more than 1479.8 Hz, the output voltage of the sensor remains basically unchanged.

**Figure 11:**
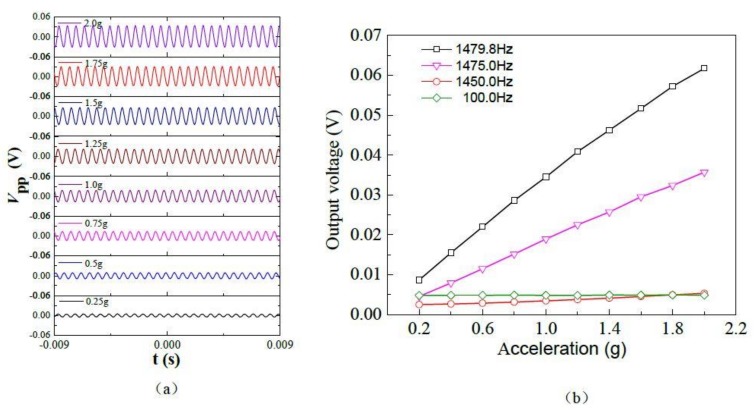
Piezoelectric characteristics curves of the sensor under different accelerations: (**a**) the output voltage waveforms of piezoelectric response; (**b**) the relationship curves between output voltage and *a*.

According to the testing results of the dynamic characteristics, an excitation signal is controlled by the software to the standard vibration table through the power amplifier, the testing module generates acceleration *a*, which travels along the *z*-axis direction, and the cantilever beam is deformed elastically, we can observe the piezoelectric response outputs’ voltage via an oscilloscope. Under different *a*, the piezoelectric response output voltage waveforms as shown in Figure 11a. The experimental results show that the voltage peak-to-peak value (*V*_pp_) of the piezoelectric response output voltage waveform increases with acceleration value. When *a* increases to 2.0 g, the *V*_pp_ reaches its maximum. Based on the above research, we further studied the relationship between output voltage and acceleration at a fixed frequency. Figure 11b shows the relationship curves between output voltage and *a* when the excitation frequency is at 1479.8 Hz, 1475.0 Hz, 1450.0 Hz, 100.0 Hz. When the excitation frequency is 1479.8 Hz, the sensitive characteristics of the proposed sensor are stronger. When the excitation frequency is 100.0 Hz, the sensitive characteristics are weak. The results show that the proposed sensor based on Li-doped ZnO piezoelectric thin films can achieve the acceleration measurement. According to the Equation (6), we calculate that the sensitivity of the acceleration sensor reaches 29.48 mV/g at resonant frequency. 

In recent years, ZnO thin films were prepared by different technology methods, such as the RF magnetron sputtering method and the sol-gel method, etc. Table 1 given compares of preparation method, the piezoelectric coefficient and the application for ZnO thin films. In this study, using MEMS technology, the acceleration sensor with a Li-doped ZnO thin films piezoelectric structure was fabricated and packaged on a silicon substrate. The results show that the proposed sensor can be applied to the acceleration measurement.

## 5. Conclusions

In this paper, the acceleration sensor with Li-doped ZnO piezoelectric thin films is fabricated and packaged by MEMS technology. Via XRD, XPS, SEM, and PFM, we analysis the influence of sputtering power and the Li-doping concentration on the microstructure of deposited ZnO thin films by using the RF magnetron sputtering method. The analysis shows that a Li-doping concentration of 5 wt% is the most appropriate, meanwhile, when the sputtering power is 220 W, the grain uniformity of the films is better, and there are no obvious holes, gaps, and wrinkles. Through the characteristics test of sensor, the experimental results show that can achieve the acceleration measurement and the sensitivity of the proposed sensor is 29.48 mV/g in range from 0 g to 2 g.

## Figures and Tables

**Table 1:** The preparation method, piezoelectric coefficient, and application of the ZnO piezoelectric thin films.

Materials	Preparation Method	Piezoelectric Coefficient and Application	Reference
Li-doped ZnO	Sol-gel method	*d*_31_ = 1.50 nm/V	[8]
ZnO	RF magnetron sputtering method	*d*_33_ = 12.30 pm/V Application: piezopolymeric transducer	[9]
ZnO		*d*_31_ = −3.21 pC/N Application: vibration energy harvesting Resonant frequency: 24,988 Hz Output power: 0.98 μW	[19]
ZnO		*d*_31_ = 3.32 pC/N Application: energy scavenger	[20]
ZnO		Application: accelerometer Sensitivity:1.69 mV/g	[21]
ZnO	RF magnetron sputtering method	Application: accelerometer Sensitivity: 44.70 fC/g (mV/g) at resonant frequency (1.02 kHz)	[22]
Li-doped ZnO	RF magnetron sputtering method	*d*_33_ = 10.55 pm/V Application: acceleration sensor Sensitivity: 29.48 mV/g at resonant frequency (1479.8 Hz)	In this work
